# Bacteriophage-Derived Depolymerases against Bacterial Biofilm

**DOI:** 10.3390/antibiotics10020175

**Published:** 2021-02-10

**Authors:** Gracja Topka-Bielecka, Aleksandra Dydecka, Agnieszka Necel, Sylwia Bloch, Bożena Nejman-Faleńczyk, Grzegorz Węgrzyn, Alicja Węgrzyn

**Affiliations:** 1 Department of Molecular Biology, Faculty of Biology, University of Gdańsk, Wita Stwosza 59, 80-308 Gdańsk, Poland; gracja.topka@phdstud.ug.edu.pl (G.T.-B.); aleksandra.dydecka@phdstud.ug.edu.pl (A.D.); agnieszka.necel@phdstud.ug.edu.pl (A.N.); bozena.nejman-falenczyk@ug.edu.pl (B.N.-F.); grzegorz.wegrzyn@biol.ug.edu.pl (G.W.); 2 Laboratory of Phage Therapy, Institute of Biochemistry and Biophysics, Polish Academy of Sciences, Kładki 24, 80-822 Gdańsk, Poland; sylwia.bloch@ug.edu.pl

**Keywords:** bacterial biofilm, bacteriophages, phage-encoded depolymerases, combined therapy

## Abstract

In addition to specific antibiotic resistance, the formation of bacterial biofilm causes another level of complications in attempts to eradicate pathogenic or harmful bacteria, including difficult penetration of drugs through biofilm structures to bacterial cells, impairment of immunological response of the host, and accumulation of various bioactive compounds (enzymes and others) affecting host physiology and changing local pH values, which further influence various biological functions. In this review article, we provide an overview on the formation of bacterial biofilm and its properties, and then we focus on the possible use of phage-derived depolymerases to combat bacterial cells included in this complex structure. On the basis of the literature review, we conclude that, although these bacteriophage-encoded enzymes may be effective in destroying specific compounds involved in the formation of biofilm, they are rarely sufficient to eradicate all bacterial cells. Nevertheless, a combined therapy, employing depolymerases together with antibiotics and/or other antibacterial agents or factors, may provide an effective approach to treat infections caused by bacteria able to form biofilms.

## 1. Introduction

Development of multidrug resistance by bacteria is an extremely serious problem in medicine and veterinary [1]. Infections by bacteria resistant to most or even all known antibiotics cause severe diseases, characterized by high morbidity and mortality (summarized in [1,2]). Therefore, development of novel therapeutic approaches is recognized as one of priorities in the era of the “antibiotic resistance crisis” [2].

Contrary to early thoughts on bacterial life, these prokaryotic organisms not only occur in a planktonic form, but can also form higher-order structures, called biofilms [3]. As in the case of any other biological processes, one can identify positive and negative aspects of biofilm formation for human life. For example, bacterial biofilms are crucial for effective functions of microbial fuel cells, for efficient production of various products during fermentation, and for biological stages of wastewater treatment [4]. However, pathogenic bacteria can form biofilms on surfaces of various materials used in medicine, as well as on surfaces of patients’ tissues [5]. Importantly, the formation of biofilm causes further resistance of bacteria to antibiotics, even if the same bacteria are susceptible to them when occurring in a planktonic form. As indicated recently, even plastic litter can be used as a surface for accumulation of pathogenic bacteria in the form of a biofilm and development of multidrug resistance [6]. Therefore, finding of effective approaches to combat bacteria included in biofilm is an important issue.

In this review article, we present an overview on bacterial biofilms, and we focus on the use of bacteriophage-derived depolymerases as potentially effective agents to destroy these structures and to facilitate antibacterial actions of other compounds or factors.

## 2. Characterization of Bacterial Biofilms

In 1933, Henrici observed that water bacteria firmly adherent to submerged surfaces create a deposit of bacteria [7]. Over time, it was revealed as a biofilm. In 1975, Mack et al. [8] described this structure for the first time as bacterial communities.

A biofilm is a community of microorganisms associated with a surface or adherent to one another and living within an extracellular polymeric exopolysaccharide matrix. Production of extracellular polymeric substances (EPSs) occurs during the attachment stage of a biofilm to the surface. EPSs provide the binding strength of microorganisms in the biofilm.

**Figure 1:**
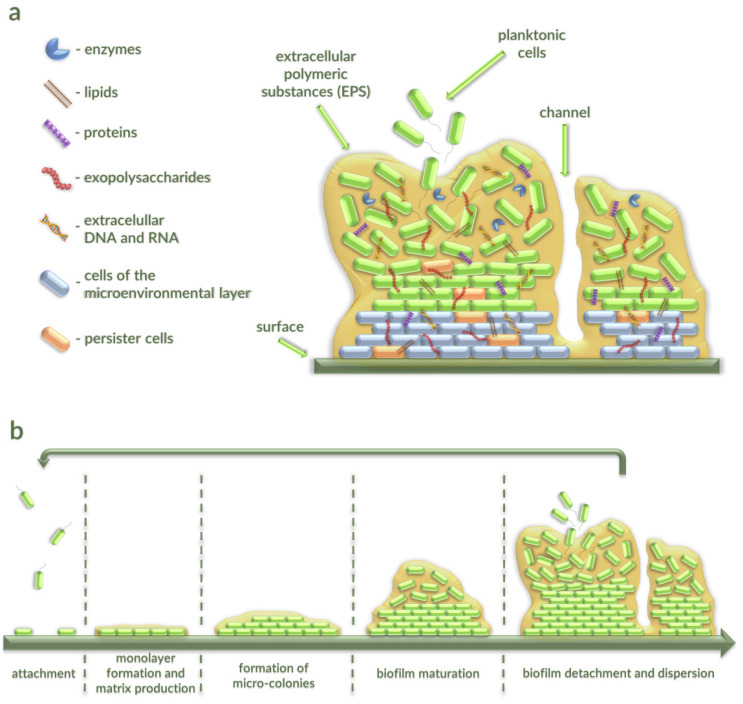
Schematic representation of biofilm components (**a**) and life cycle (**b**). (**a**) The mature biofilm is built with a variety of compounds (DNA, RNA, proteins, lipids, enzymes, and extracellular polysaccharides) called extracellular polymeric substances (EPSs). (**b**) Formation of biofilm starts with attachment of planktonic cells to the surface. Next, bacteria start to form a monolayer and produce the matrix which allows developing the mature biofilm. In the last stage, bacterial cells multiplicate quickly, start to detach, and disperse. This process enables them to convert to motile forms that can spread and colonize new surfaces.

EPSs consist of DNA (<1%), RNA (<1%), structural proteins (1–60%), lipids (1–40%), enzymes, and one or more of extracellular polysaccharides (40–95%), where polymers composed of sugar residues are secreted by bacteria into the surrounding environment [9,10,11] (Figure 1a).

These substances protect bacteria from predation and a variety of physical, chemical, and biological factors, and help them to survive in hostile environments [12]. The remaining volume of biofilm (typically 2–35%) is constituted by the microorganisms themselves. Channels in the biofilm allow getting water, air, and nutrients to all parts of this structure [13]. Particular layers of microorganisms in biofilms bind to existing bacteria through coaggregation [14].

### 2.1. Stages of Bacterial Biofilm Formation

Biofilm formation is a complex process during which intercellular and intracellular signaling occur. This process consists of five stages (Figure 1b). The first is the microorganism’s attachment to a living or nonliving surface with the use van der Waals forces, as well as by employing fimbriae and flagella. In the next step, bacteria form a monolayer and anchor themselves by producing an extracellular matrix. Next, the process of multiplication and division of microbial cells starts, initiated through particular chemical signaling within the EPSs. This action leads to the formation of microcolonies and gives rise to three-dimensional structures [9]. In the following phase, a thin biofilm is formed from layered cells and small clusters. As a result, the maturation and formation of the architecture of the biofilm take place. Clusters develop into large microcolonies, and many cells displace from the substratum to form channels and voids [15]. In the last stage of biofilm formation, microbial cells upregulate the expression of genes coding for proteins related to flagella formation, allowing the bacteria to move to a new location. Bacteria within the biofilm start to multiplicate quickly, detach, and disperse. This process enables conversion of bacteria to a motile form, with subsequent spreading and colonization of new surfaces [9]. The release of cells from a biofilm may be caused by different events, including environmental cues and microorganism-derived signals. On the one hand, the active bacterial escape from the protective biofilm environment is associated with nutrient deprivation, steepening concentration gradients of oxygen and waste products, and extracellular signaling compounds over the course of biofilm development that lead to the stress response and accumulation of molecules that are capable of inducing dispersion. Importantly, sensing and relay of signal is performed via a series of post-transcriptional modifications that result in modulation of cyclic di-guanosine monophosphate (c-di-GMP) level. A low concentration of this intracellular signaling molecule enhances motility and the planktonic mode of growth [16]. In turn, the passive release of cells or their loss is induced by mechanical, physical, or frictional forces. Notably, layers of biofilm may be broken off due to (i) natural collision of biofilm cells with environmental particles (abrasion), (ii) feeding activity of eukaryotic organisms (grazing), or (iii) frictional forces caused by the velocity of the liquids in the aqueous environment (erosion and sloughing) [16,17].

### 2.2. The Occurence of Bacterial Biofilms

Bacterial biofilms are capable of adhering to a wide variety of surfaces, both biotic and abiotic, which can be observed in every natural environment, such as streams, lakes, and oceans, as well as on medical devices and in human tissues (for example, teeth or heart valves) [18]. Recent investigations suggested the occurrence of biofilms in extreme environments, such as acid mines (pH close to 0), thermal springs, and the “desert-like” lake ice cover of Lake Bonney, Antarctica [10]. Due to their widespread occurrence, biofilms represent a major threat causing infectious diseases and economic losses. The National Institutes of Health (NIH) revealed that over 80% of microbial infections in the body are associated with biofilms (program announcement PA-03-047) [16]. The formation of biofilms in healthcare settings is extremely problematic. Biofilm has been found on medical devices and prostheses, water lines and tubing, endoscopes, intravenous and urinary catheters, and wounds [19,20,21]. From these surfaces, microbes can readily spread to patients and cause acute infections. 

Biofilms may be composed of only a single or of different types of microbial species. Interestingly, microorganisms which are attached to dental plaque potentially leading to dental caries consist of *Pseudomonas aerobicus* and *Fusobacterium nucleatum*. They are the main agents of gingivitis and periodontitis [22]. Moreover, contact lenses are mainly colonized by *Escherichia coli*, *Pseudomonas aeruginosa*, *Staphylococcus aureus*, *Staphylococcus epidermidis*, *Serratia* spp., and *Proteus* spp. bacteria and various species of the *Candida* genus. Additionally, venous catheters can be contaminated by *P. aeruginosa*, *Enterobacter* spp., and *Klebsiella* spp., while urinary catheters can be contaminated by *E. coli*, *Enterococcus faecalis*, *S. epidermidis*, *P. aeruginosa*, *Proteus mirabilis*, *Klebsiella pneumoniae*, and other Gram-negative bacteria [9].

Examples of biofilm-associated infections also include lung infections in cystic fibrosis, caused by *P. aeruginosa*, eventually leading to lung failure [23]. Biofilms are also the root of many problems in the food industry. Microbes that occur in food preparation and water distribution systems can result in contamination of food products, causing persistent and chronic bacterial foodborne infections. Pathogens such as *E. coli*, *Salmonella* spp., *Shigella* spp., *S. aureus*, *Vibrio* spp., *Campylobacter jejuni*, *Clostridium* spp., and *Listeria monocytogenes* form biofilms in the food processing environment [15].

### 2.3. Antibiotic Resistance in Bacterial Biofilm

Biofilm formation imposes a serious threat for human health worldwide. Antibiotic resistance of bacteria in biofilm communities contributes to serious infections. Bacteria in biofilms reveal about a 1000-fold decrease in susceptibility to antibiotics compared with planktonic cells [24,25,26].

Currently, it is believed that over 80% of chronic infectious diseases are caused by biofilms, and it is known that conventional antibiotic medications are inadequate at eradicating these biofilm-mediated infections [5,27]. The spreads of biofilm-related infections cause an intractable problem in modern medicine [28]. Regardless of their location, bacteria in biofilms are tolerant or resistant to the response of the host immune system, antibiotic therapy, antiseptic agents, or disinfectants and germicides [29,30].

**Figure 2:**
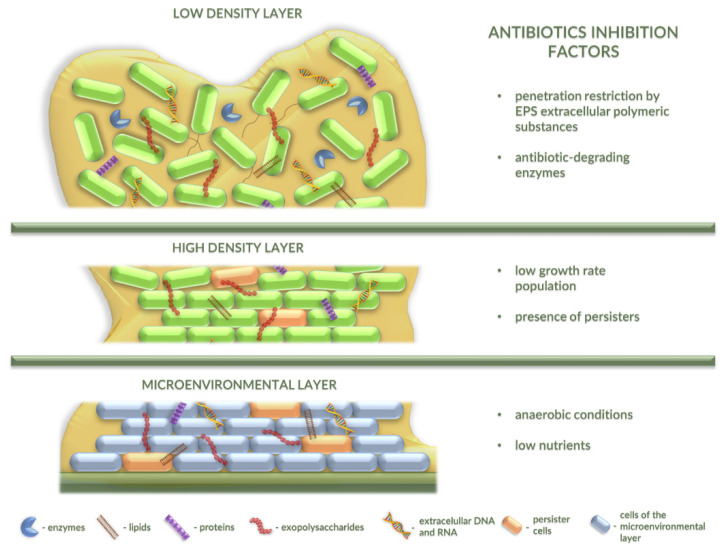
Bacterial biofilm layers and factors that can inhibit activities of antibiotics. Mature biofilm may be divided into three layers: low-density layer, high-density layer, and microenvironmental layer with slow growth and persister cells. All of the layers contain anti-antibiotic factors and can resist antibiotic activity in different ways.

It is worth mentioning that the tolerance of bacterial biofilms to antimicrobials is multifactorial and is based on different molecular strategies of protection of bacterial cells from hostile conditions. Difficulties in controlling biofilm formation arise from intrinsic and acquired resistance mechanisms of microorganisms. As reported, the resistance of microorganisms in a biofilm may be related to (i) interaction of the biofilm matrix with antibiotics that can retard and lower their activities, (ii) slow growth rates of bacteria in which drugs are not effective, (iii) genetic changes of target cells or hiding the target sites, (iv) action of the modifying enzymes, (v) generation of persister cells which are tolerant to different antibiotics, (vi) alteration of the chemical microenvironment, (vii) multiple microbial species, and (viii) the age of the biofilm (Figure 2). Thus, this multifactorial nature of bacterial biofilms with respect to antimicrobial tolerance is a great challenge for the use of conventional antibiofilm therapeutic strategies [5,31].

The bacterial resistance is related to the biofilm matrix structure which protects bacteria from a variety of physical, chemical, and biological factors, and which inactivates or impairs antimicrobial molecule spread through the polymer matrix [31]. Due to the impermeability of the biofilm matrix and the diversity of bacterial cells within this structure, antibiotics usually fail when treating biofilm-related infections [32,33]. Therefore, these diseases often tend to be recurring, even when formed by opportunistic bacterial microorganisms [34]. In many cases, the use of antibiotics, such as colistin, imipenem, fluoroquinolones, beta-lactams, aminoglycosides, and others, can only reduce the biofilm layers but cannot eliminate them completely [31,35,36]. 

EPSs hold bacterial cells together and lead to the development of multicellular consortia, allowing the biofilm to function as a multicellular system. The formation of these biofilm complex structures is often regulated by the communication between bacterial cells in the quorum sensing (QS) process that enables the bacterial community to survive environmental stresses and actions of antimicrobial agents [12,37,38]. The presence of antibiotic-tolerant subpopulations has been confirmed in biofilms formed by various bacterial species. Chua et al. observed development of colistin-tolerant subpopulations in biofilms formed by *P. aeruginosa*. Notably, colistin-tolerant cells were able to migrate toward the dead microcolonies of the antibiotic-treated biofilm using type IV pili, where they initiated formation of new biofilm via QS [39]. Importantly, the components of the QS system have been identified as genetic determinants responsible for formation of biofilm in the presence of antibiotics by *E. faecalis* and other bacterial species [40]. On the other hand, inhibition of QS system was shown to increase the susceptibility of *S. aureus* biofilms toward different classes of antibiotics [41]. Undoubtedly, multicellular behaviors such as migration and cell–cell signaling seem to play an important role in bacterial biofilm formation, life cycle, and resistance to antibiotics and other antimicrobial agents.

Interestingly, the high density of the EPS matrix and its binding properties to antimicrobial agents build an effective barrier that can restrict diffusion of antibiotics to various layers of the biofilm [29]. Additionally, toxic compounds can interact with this polymeric matrix and decrease its activity through enzymatic reactions or chelation of the antibiotics [42,43]. It is commonly known that alginates are able to block the diffusion of gentamicin or tobramycin. Moreover, the exopolysaccharides can protect biofilms of *P. aeruginosa* from aminoglycosides by directly binding these cationic antibiotics [44].

The persister cells form a small subpopulation of slow-growing or growth-arrested bacteria. Their occurrence in the biofilm structure is related to poor diffusion of nutrients and oxygen into periphery region of the biofilm. Interestingly, the persister cells become highly tolerant to antibiotics. This state of resistance is not achieved by genetic changes [45]. It is proposed that this phenomenon relates to the lack of active targets that antibiotics can corrupt (i.e., no replication of persister cells and slow death) [27]. Importantly, persister cells represent a small fraction (0.1–10%) of the entire population of biofilms formed by *P. aeruginosa*, *E. coli*, *S. aureus*, *Acinetobacter* spp., *Salmonella* spp., and other bacteria [45]; however, they can survive the presence of 1000-fold the minimum inhibitory concentration of various antibiotics [32]. Therefore, persister cells appear to be responsible for the inability to eliminate chronic infection by using antibiotic treatment [46].

The antibiotic resistance of biofilms may also be conditioned by the presence of bacterial cells carrying resistance genes encoding enzymes, such as, e.g., β-lactamases or aminoglycoside adenylyltransferases, which can inactivate or modify antimicrobial agents. Such enzymes are secreted into the biofilm matrix and prevent these agents from reaching their cellular nonresistant targets [29,47]. For instance, *K. pneumoniae* biofilms produce and secret β-lactamase that was found to effectively degrade ampicillin and prevent it from reaching other cells within the biofilm [47]. In turn, genes encoding aminoglycoside-modifying enzymes have been found in *E. coli*, *Salmonella enterica*, and *P. aeruginosa.* They occur in a high number of variants, and their products can utilize aminoglycoside antibiotics as substrates [48]. In effect, products of antibiotic-resistant genes expressed in some bacterial cells in biofilm can protect other nonresistant bacteria against the action of antibiotics. Importantly, cells carrying such genes are resistant to antibiotics, irrespective of whether they are present in a biofilm or not. 

It is worth mentioning that antibiotic tolerance in the bacterial biofilms may also be related to oxygen limitation in the lower layers of its structure. Antibiotic resistance in this system is likely due to the fact that anaerobic conditions restrict bacterial metabolic activity to a narrow zone adjacent to the air interface. Interestingly, outside this zone, bacteria included in the biofilm are not easily killed by antibiotics. Importantly, an oxygen gradient is a common feature of life in biofilm, and such conditions may be correlated with aminoglycoside and fluoroquinolone tolerance of the *P. aeruginosa* biofilm system [49].

Moreover, high-density biofilms also display steep gradients of nutrient availability from the periphery of the biofilm to the center. This phenomenon leads to metabolic dormancy of the majority of the bacterial community located toward its interior. Such metabolically repressed antibiotic-tolerant cells are specific for biofilm-producing *P. aeruginosa* [23,50].

Nearly all biofilm communities in natural environments contain a variety of different bacterial species. Importantly, due to cooperative interactions between them, the multispecies biofilms may display greater resistance to external stressful factors, including antibiotics and disinfectants, relative to the single-species community. This property is probably related to increased biomass and/or an altered composition of the EPS matrix [51,52,53].

Importantly, the age of bacterial biofilm may have also an impact on the effectiveness of the action of antibacterial agents. Many biofilm communities enter into the stationary phase with time, suggesting that older biofilms show higher tolerance to antibiotics. Interestingly, Chen and coworkers reported that the mature biofilms formed by *P. aeruginosa* or *S. aureus* were more difficult to eradicate by using different antibiotics than younger ones. This is probably due to differences in biofilm structure, differences in EPS matrix composition, and/or phenotypical changes [47,54].

### 2.4. Alternative Strategies for Combating Bacterial Biofilms

**Figure 3:**
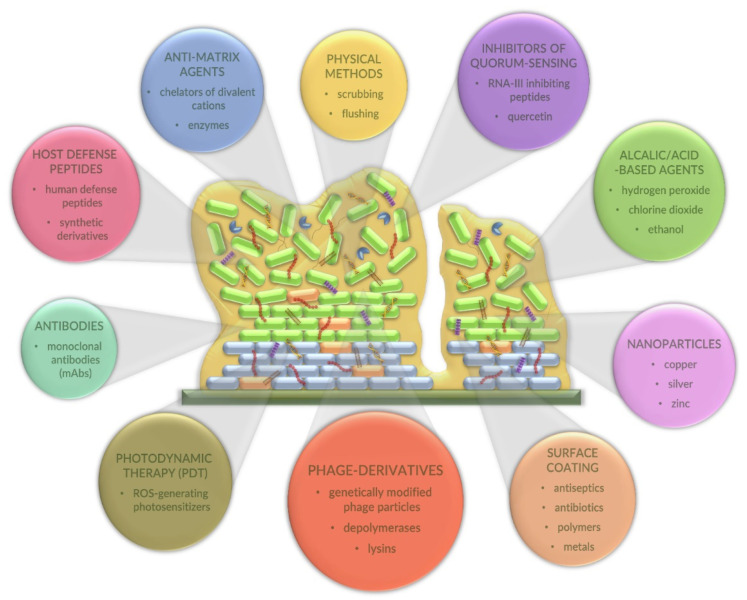
Alternative strategies in the fight against mature biofilm or its formation. Circles show the diverse antibiofilm agents with examples provided. The most common way of treating biofilm infection is the disposal of the contaminated device (e.g., medical equipment implanted) or the removal of formed biofilm [33].

So far, several alternative strategies (different than antibiotic therapy) to combat biofilms have been proposed (Figure 3).

One such strategy is based on surface modification or coating methods which lead to difficulties of microorganism attachment. By using the materials resistant to microbial adhesion and incorporating antimicrobial agents (antiseptics, antibiotics, or metals) onto the surface, biofilm formation is impeded [15,30,55]. This approach may be exemplified by interesting applications in medicine where bacterial infections associated with medical devices state a significant problem. Antibiofilm metal-based coatings have been developed using silver, titanium, zinc, and copper ions. Metals are applied in order to destroy bacterial integrity and to prevent the proliferation of bacteria on the surface of various medical devices [56]. The main limitation of the metal-based strategy is the concentration of metallic ions, as it should not induce cytotoxic effects. To overcome this primary limitation, different capping agents, e.g., Kocuran, polyethylene glycol (PEG)-heparin hydrogels, and β-cyclodextrin (β-CD), have been applied [56]. In another coating approach, Kart and collaborators [57] showed that nitrofurazone-impregnated catheters significantly reduced the biofilm cell counts of *E. faecalis* and completely inhibited the biofilm-forming ability of *P. aeruginosa* and *S. epidermis*. Interestingly, in comparison to hydrophilic or silver-coated catheters, nitrofurazone-impregnated tubes showed more prolonged antimicrobial durability [57]. Another example of a surface-coating material is bioactive glass. Marques et al. presented that titanium implants coated with F18 bioactive glass (F18BG) had improved antibiofilm activity in comparison to titanium, especially in the initial periods of biofilm formation by *Candida albicans*, *P. aeruginosa*, and *S. epidermidis* [58]. Recent studies also indicated the application of bioactive glass as a vehicle for the controlled release of therapeutic molecules [59]. Pillararene-based multilayers used for the delivery and release of antibiotics showed similar properties. As indicated, the use of decacarboxylato-pillar(5)arene/poly(allylaminehydrochloride) 8 ((WP5/PAH)8) multilayered films loaded with levofloxacin and amikacin reduced the adhesion and proliferation of *P. aeruginosa* and *S. aureus* cells [60]. Undoubtedly, the use of surfaces capable of preventing or reducing bacterial biofilm formation is one of the important strategies in the fight against bacterial biofilms, especially applicable in the fabrication of medical equipment. It is difficult to indicate their superiority over antibiotics or phage-derived depolymerases, as the coating materials serve frequently as multiple drug reservoirs able to release antibacterial agents.

Interestingly, direct action on the matrix components, using enzymes, such as deoxyribonuclease I or dispersin B [61], chelators of divalent cations [62], or usnic acid reducing various sugars in EPS, can be classified as an alternative method in the fight against bacterial biofilm upon its establishment [63].

It is worth mentioning that naturally occurring host defense (antimicrobial) peptides (HDPs), or their synthetic derivatives, also represent an appealing source for generation of promising therapeutic agents for the treatment of persistent infection caused by biofilms formed by drug-resistant Gram-negative and Gram–positive bacteria, such as *E. faecium*, *S. aureus*, *K. pneumoniae*, *A. baumannii*, *P. aeruginosa*, *E. coli*, *L. monocytogenes*, and *S. enterica* [64,65]. Importantly, these small molecules are highly efficacious, are generally nontoxic for mammalian cells, and show broad-spectrum antibacterial activity via employment of sophisticated and dynamic mechanisms of action [66,67,68]. Moreover, HDPs are able to effective combat biofilms because of their ability to penetrate, derange, and disperse biofilm structures [69]. One of the best-characterized representatives of natural HDPs is the human defense peptide LL-37 that affected biofilm formation of *P. aeruginosa* [70]. On the other hand, the following molecules belong to the group of synthetic peptides with efficient antibiofilm activity: peptide 1037 (LL-37 derivative), peptide 1018 (bactenecin derivative), NRC16 (pleurocidin derivative), P10 (P60.4Ac derivative), a hybrid peptide CAMA (cecropin-A and melittin-A derivative), and others [64]. Furthermore, some HDPs synergize with the action of antibiotics, which leads to the repression of molecular pathways regulating biofilm development [64,69]. This kind of combinatorial approach, based on antimicrobial peptides and conventional antibiotics, has been applied successfully against biofilms formed by *P. aeruginosa*, *E. coli, A. baumannii*, *K. pneumoniae*, *S. enterica*, and methicillin-resistant *S. aureus* [64]. Additionally, such combination therapy is also applicable to biomedicine, an example of which is the synergistic effect of cryptic peptides of human apolipoprotein B and ciprofloxacin against *Pseudomonas* and *Burkholderia* strains, clinically isolated from patients with cystic fibrosis [68]. It is worth emphasizing that the greatest advantage of this combination therapy is the reduction of the selective pressure for the development of drug resistance, as it decreases concentrations of both used agents, i.e., peptides and antibiotics [64,68].

The role of QS in biofilm formation has been demonstrated in many bacterial species. These systems influence the heterogeneous architecture of bacterial biofilms in the regulation of the synthesis of the degradative enzymes. QS may also regulate the antibiotic susceptibility by increasing antibiotic tolerance in bacterial biofilms [65]. Interestingly, QS deficiency can be associated with the formation of thinner biofilm layers and lower EPS production, which leads to the kanamycin sensitivity of biofilms of *P. aeruginosa* [71,72]. Consequently, QS is considered a promising target for new antibiofilm agents. Moreover, QS inhibitors (enzymes and auto-inducing peptides) or RNA-III-inhibiting peptides [65], halogenated furanone compounds [73], quercetin [74], curcumin [33], and even ginseng extract or garlic extract [75,76] contribute to overcoming the bacterial resistance in biofilm.

Antimicrobial peptides that can inhibit bacterial cell division, such as pyrrhocoricin or microcin B17, also belong to the group of anti-biofilm agents. Pyrrhocoricin is capable of binding with the multi-helical lid region of the bacterial heat-shock protein DnaK, and it interferes with the initiation step of DNA replication. Additionally, pyrrhocoricin takes part in the interaction between DnaK and DnaJ that causes cell death. This proline-rich antibacterial peptide is also responsible for disrupting the translation process by binding to the tunnel of the ribosome. Interestingly, microcin B17 may also inhibit the replication process of bacterial DNA by interacting with DNA gyrase [33].

Interestingly, Hook and coworkers identified a group of structurally related materials comprising ester and cyclic hydrocarbon moieties that are able to reduce the attachment of pathogenic bacteria, such as *P. aeruginosa*, *S. aureus*, and *E. coli* [12,77]. Importantly, this bacterial–material interaction is strictly dependent on surface chemistry and can be related to the individual cells sensing the nature of the polymer surface via sensory proteins of the bacterial envelope or a specific surface structure such as flagella and pili. It is also speculated that the anti-attachment bacterial response to the presence of the polymer may be regulated by bacterial cell-to-cell communication through QS mechanisms [6,12].

An alternative approach to combat bacterial biofilms is to reduce formation of persisters that are one of the main barriers for the effectiveness of antibiotic therapy. These dormant cells are protected by the biofilm matrix and have probably disabled their programmed cell death (PCD) to allow survival of a few cells if the antimicrobial agent reaches the whole population [29,78]. Therefore, a novel anti-persister strategy is associated with the inhibition of the (p)ppGpp-regulated stringent response that is probably the main signal leading to dormant cell formation [32,79]. It has also been shown that silver, halogenated indoles, and the 1018-peptide may increase antibiotic sensitivity and attenuate biofilm virulence [80,81,82]. Undoubtedly, all factors affecting persister cells are desirable because they decrease the multidrug tolerance of the biofilm.

Antibiofilm strategies based on nanotechnology are also noteworthy. These methods include a wide range of metal-based nanoparticles (silver, gold, titanium, zinc, or copper), polymer-based nanoparticles, and green nanoparticles that are known for their antimicrobial action [30,37,83] It is well known that silver has been widely employed as an antimicrobial agent with a broad spectrum of activity against bacterial biofilms. The nanoparticles made of silver have been shown to interact with bacterial membrane proteins, intracellular proteins, and phosphate residues in DNA, as well as to interfere with cell division, leading to bacterial death [84]. The toxicity of silver nanoparticles is a serious problem that limits their use to certain sites. Oxidative stress, DNA damage, and cytokine induction have been proposed as three main mechanisms responsible for cytotoxic effects [85].

Disinfection methods are also able to disrupt the biofilm. They are based on the cleaning of medical and industrial equipment by using chemical alkali-based and acid-based agents, ethanol, chlorine dioxide, or hydrogen peroxide, coupled with physical methods, such as scrubbing and flushing [15].

Importantly, the activity of phages against biofilm is also exploited. This strategy is based on single phages or phage cocktails, phage-derived enzymes, phages in combination with antibiotics, and genetically modified phages [33,86,87]. Although phages depend upon a metabolically active bacterial host to replicate, and they are generally unable to replicate in dormant/persister cells until these are reactivated, one might suggest an advantage of using these viruses against biofilms due to their lytic activity against bacteria with reduced (although not completely inhibited) metabolism. Moreover, although most phages can be affected by the EPS matrix, they remain able to disseminate within the biofilm and replicate, at least to some extent. Importantly, bacteriophages are not motile agents, and they should preferably be added directly to the biofilm. They present high specificity against bacterial species and even strains. Furthermore, the development of bacteriophage resistance is another concern that arises in the case of phage therapy [88].

The use of monoclonal antibodies (mAbs) is an another interesting antibiofilm strategy. Tursi et al. presented human mAbs that could bind the bacterial amyloid curli and inhibit curli monomer polymerization, thus disrupting biofilm formed by *S. enterica* serovar Typhimurium. Biofilm treated in this way displayed changes in ECM composition and a “loose” structure. Moreover, injection of the human mAbs facilitated the antibiotic-mediated eradication of biofilms formed on a glass slide and vascular catheter [89]. As indicated previously, antibody-based approaches can be used to combat biofilms of various bacterial species, such as *S. aureus*, *A. baumannii* [90], or *S. epidermidis* [91]. Additionally, they can be successfully applied as a device-coating strategy and in combination with antibiotics. Importantly, in contrast to antibiotics, mAbs do not cause resistance development among bacteria, present a high safety level, and have few off-target effects [89].

Another strategy that has gained increasing attention in the treatment of bacterial biofilm-associated infections is antimicrobial photodynamic therapy (PDT). This promising approach, based on the delivery of light-activated photosensitizers (PSs) that generate cytotoxic species, such as ROS (reactive oxygen species), has been reported by different authors. Quite recently, Zagami and collaborators showed photobacterial activity of sulfobutylether-β-cyclodextrin/cationic porphyrin *meso*-tetra(4-*N*-methylpyridyl)porphine (TMPyP) nanoassemblies against *P. aeruginosa, E. coli*, and *S. aureus,* pathogens frequently involved in biofilm-related infections [92]. In turn, Yuan and collaborators developed a synergistic photothermal (PTT)/photodynamic (PDT) therapy strategy for combating the biofilm formed by *S. aureus* on a titanium implant [93]. Interestingly, the superiority of this combined PTT/PDT strategy over PTT alone is related to the temperature, which is lower, thus enhancing the antibacterial effect [94]. On the other hand, the important advantage of using PDT alone or in the combination strategy is the lack of resistant mutants which may occur in other approaches, including antibiotic therapy.

The use of the above-described strategies depends on whether they are intended to prevent or treat nosocomial infections. It is also important to assess whether the biofilm is associated with medical devices or formed on native host tissues. In addition to infections associated with insertion of implants, prostheses, tubes, catheters, and other devices, biofilms are involved in causing endocarditis, urinary tract infections, sinusitis, colitis, dental plaques, gingivitis, and chronic wound infections [29,95,96]. Unfortunately, not all of the described strategies can be used to treat infections associated with the formation of biofilm in the patient’s body. For example, disinfection or surface-coating techniques are used to prevent medical device-associated infections, but not to treat them. The use of antibiotics is an alternative, although they must be administered at very high concentrations and over a long period of time. Unfortunately, antibiotics and other conventional strategies have limited activity against persisters. Sometimes, surgical removal of the source of biofilm is the only effective solution. As indicated above, it was proposed to use bacteriophages to treat biofilm infections. Several preclinical animal studies supporting the application of phages in these kinds of infections were described and analyzed by Doub in a recent review paper [88]. Importantly, the synergistic activity of phages with antibiotics is also observed. Recently, the quality of phage preparations, their titer and dosage, their routes of administration (local or intravenous), and their safety and interactions with eukaryotic cells have been evaluated and widely discussed in the literature [88,97]. In contrast to the treatment based on the use of whole viral particles, the application of phage-derived depolymerases may overcome these difficulties. Thus, their use against bacterial biofilms is broadly considered below.

## 3. Bacteriophage Depolymerases as an Alternative to Antibiotics

Phage depolymerases have gained the interest of the scientific world due to their participation in phage adsorption and digestion of bacterial capsules. According to Pires et al., most of these proteins are encoded in the region of structural genes in a phage genome (i.e., tail fibers and base plates) or next to it [98]. These phage-encoded enzymes recognize, bind, and digest the polysaccharide compounds of bacterial cell walls [80]. Degradation of these structures allows exposing the phage receptor, which is crucial for efficient phage infection of the host [99]. Bearing in mind the mechanism of action of phage depolymerases, we can divide them into two classes, hydrolases and lyases. Both groups are focused on the process of degradation of polysaccharides, including capsular polysaccharides (CPSs), lipopolysaccharides (LPSs), *O*-polysaccharides, or exopolysaccharides (EPSs), produced in the biofilm [81]. Some of them can also degrade polypeptides or lipids [100]. Interestingly, phage hydrolases mainly have the activities of sialidases, xylosidases, levanases, rhamnosidases, dextranases, and peptidases. This group of enzymes belongs to the *O*-glycosyl hydrolases that catalyze the hydrolysis of glycosidic bonds [98,99]. Moreover, another class of depolymerases is represented by hyaluronate, alginate, and pectin/pectate lyases. Their mechanisms of depolymerization do not include water usage and are based on β-elimination to form a new double bond [101].

Since the discovery of activities of phage depolymerases, they have become an alternative to antibiotics, especially in the treatment of multidrug-resistant bacteria. Unlike antibiotics, depolymerases can be specific to the host and allow for the natural bacterial flora to remain untouched [102,103]. They can not only be engineered to increase their degrading activity, but also used as tools for the detection of bacteria [104]. Depolymerases may be used in two alternative forms, (i) as tail-spike proteins (TSP) which have depolymerase domains, being components of virions, or (ii) as free enzymes [101]. When whole phages are used, they can multiply in sensitive host cells, producing significantly more virions with TSPs. This option might be beneficial if there are difficulties in supplying materials directly to biofilms, e.g., during treatment of clinical biofilm infections, as phages may allow for more effective delivery of these enzymes to the target place [105]. On the other hand, phages can transfer genes coding for toxins and antibiotic resistance proteins between bacterial cells, which causes safety issues [106,107,108]. The development of bacteriophage resistance among bacteria is another problem [109,110]. In contrast, free depolymerases are genetically stable agents, active in harsh external conditions. Development of resistance to these enzymes is unlikely, which is another advantage of free proteins (obtained biotechnologically as products of recombinant genes) over the use of whole phage particles [99,111,112]. In addition, the diffusion of free enzymes seems to be more rapid and effective than that of virion-associated depolymerases [105].

As described above, bacterial biofilms are formed mainly with EPSs [11,113]. Both CPS and EPS layers present large diversity in their polysaccharide content. Differences can be observed at the species and strain levels. In addition, the EPS matrix may present structural heterogeneity across individual biofilms as the physiological state of the host cells, their growth phase, their co-aggregation, and the environmental conditions also affect its composition [114]. Therefore, in some cases, a depolymerase that is able to degrade the CPS layer of a particular bacterial strain may not be capable of digesting its EPS layer. In response to the existing tremendous variation in bacterial polysaccharides, huge diversity in phage-derived depolymerases is observed. They present high specificity to a narrow range of target polysaccharides [99]. This feature greatly limits polysaccharides that can be digested by a particular depolymerase. In effect, a depolymerase originated from a particular phage may not recognize the cell-surface polysaccharide compounds of closely related bacteria or even those produced by bacteria of the same strain but growing under different conditions [105].

Furthermore, susceptibility of biofilms to phage depolymerases is dependent on the content of microorganisms and whether the biofilm is single- or multispecies. Importantly, polymicrobial systems not only are limited to different bacterial species, but may also encompass different genera and even other organisms, such as fungi. In comparison with single-species biofilms, the mixed communities are undoubtedly the dominant form in nature; however, they are more difficult to remove by phage depolymerases [52]. Furthermore, as reported by Burmolle et al., bacteria in multispecies biofilms display higher resistance to other antibacterial agents than biofilms formed by these bacterial species alone [115]. One possible reason for this is the limitation of antibiofilm agent migration by the presence of a large diversity of EPSs produced by heterogeneously distributed bacteria. In such a heterogeneous community, the action of depolymerases is limited due to the fact that they are highly specific for the host-derived polysaccharides [116]. Complex EPSs of multispecies biofilms may not only hinder the penetration of the biofilm by depolymerase-producing phages, but also entrap the phage in the biofilm matrix [117], reduce multiplication of the phage due to the presence of phage non-susceptible or metabolically inactive cells [118], reduce the presentation of the phage receptor [119], or deter the phage depolymerase activity [120]. In effect, pockets of unattainable phage-susceptible bacteria are formed, enhancing the structural heterogeneity of multispecies biofilms [116].

Importantly, there have been attempts to overcome the abovementioned difficulties in combating polymicrobial biofilm communities. One of them involves phage depolymerases, able to degrade EPSs of different bacterial species. As reported by Skillman et al., over a 90% reduction in dual-species biofilm was obtained using polysaccharide depolymerase, isolated from a bacteriophage [121]. On the other hand, multispecies biofilms can also be treated with cocktails consisting of different phages/depolymerases acting on different receptors/structures, and this strategy is even recommended [122,123] (for more examples, see review [116]). In addition, genetic modifications of phages could allow them to produce several depolymerases and extend their host range [99,114]. They have been subjected to genetic engineering and purified [114]. Importantly, free recombinant enzymes are more easily produced than virion-associated depolymerases. Moreover, such recombinant enzymes may be applied at high concentrations, which overcome enzyme production by phages alone. Currently, work is underway to expand the spectrum of their activities [124]. In the fight against biofilms (including multispecies communities), depolymerases can also be used in combination therapy with antibiotics, phages, or other agents. Examples of such combination strategies are discussed in Section 4.

### The Antibiofilm Activity of Phage Depolymerases: Examples of Applications of Phage Depolymerases against Bacterial Biofilms

EPSs are mainly responsible for the structural and functional integrity of bacterial biofilms and have an influence on their virulence [125]. Interestingly, Gutiérrez et al. [126] applied the EPS depolymerase Dpo7, derived from bacteriophage vB_SepiS-phiIPLA7, against staphylococcal biofilms. This study revealed that Dpo7 is able to degrade the EPS biofilm matrix of staphylococcal strains from 31% in *S. epidermidis* ASLD1 to 75% in *S. epidermidis* LO5081, relative to the control variants. Additionally, the pre-treatment of polystyrene surfaces with Dpo7 resulted in the reduction of biofilm activity and its biomass from 53% to 85% in the majority (67%) of tested strains (the obtained results were dose-dependent and time-independent). In summary, EPS depolymerase Dpo7 may inhibit biofilm formation and can also disperse biofilms generated by different strains of *S. epidermidis* and *S. aureus* [126].

Moreover, Hernandez-Morales et al. [127] isolated a novel bacteriophage Petty that possesses the gene of depolymerase Dpo1 (capable of degrading EPSs) and can infect *A. nosocomialis* and *A. baumannii*. The main purpose of that study was to determine the ability of Dpo1 to depolymerize EPSs and to remove bacterial biofilm formed by *Acinetobacter* strains. In vitro analyses showed that Dpo1 was able to reduce EPS viscosity and to remove biofilms of some of the tested *Acinetobacter* strains. However, the antibiofilm effect of Dpo1 was not spectacular, and it led to a 20% reduction of the bacterial biofilm. The obtained results may suggest that the phage depolymerase Dpo1 cannot completely destroy bacterial biofilms. On the other hand, Dpo1 may probably decrease the virulence of the tested bacterial strains via the degradation of EPSs [127].

In another research, Mi et al. [128] investigated the efficacy of newly isolated phage IME180 against biofilms formed by *P. aeruginosa*. This lytic phage possesses a gene that encodes a functionally active depolymerase. Surprisingly, this phage-derived enzyme is able not only to inhibit the formation of host bacterial biofilms, but also to reduce the biomass of a preformed biofilm. However, complete inhibition of biofilm formation or its removal was not observed [128].

It is worth mentioning that the ability to form biofilm is also observed in the group of *E. coli* bacteria [129]. Guo et al. [130] isolated and characterized phage vB_EcoM_ECOO78 that infects clinical isolates of *E. coli*. This phage belongs to the *Myoviridae* family, and it encodes a functionally active depolymerase Dpo42. Researchers have demonstrated that Dpo42 may degrade the capsular polysaccharides surrounding *E. coli* cells. Moreover, this enzyme can also exhibit antibiofilm activity in a dose-dependent manner. The highest level of antibiofilm activity was observed when Dpo42 was added to a final concentration of 25 μg/well. However, it was also shown that the depolymerase Dpo42 can significantly prevent biofilm formation, but cannot remove it totally [130].

In subsequent studies, depolymerase Dep6 (O91-specific polysaccharide depolymerase) was identified in the lytic T7-like phage, named PHB19, specific for Shiga toxin-producing *E. coli* (STEC). Dep6 was tested for antibiofilm and antibacterial activities against *E. coli* strains. Application of the Dep6 enzyme significantly reduced the absorbance of the total 24 h old and 48 h old biofilm biomasses, compared to untreated controls. However, Dep6 did not decrease the number of counted viable bacterial cells. Interestingly, this depolymerase may probably degrade EPS on the surface of the STEC HB10 strain, thus enhancing the susceptibility of this strain to serum killing [54].

*K. pneumoniae* has also demonstrated resistance to a wide range of antibiotics. This pathogen also belongs to the group of bacteria that are able to form biofilms [131]. Interestingly, depolymerase Dep42 was identified in lytic bacteriophage SH-KP152226 that represents the *Podoviridae* family and can lead to the lysis of *K. pneumoniae* capsular type K47 [132]. Treatment with 10 μg/mL Dep42 resulted in a reduction in bacterial counts in the biofilm, compared to the control. Wu et al. [132] also demonstrated that EPSs from *K. pneumoniae* strain 2226 can be degraded by Dep42. These results showed that depolymerase Dep42 weakly reduced the number of colonies in the biofilm but had the ability to degrade the extracellular material of the biofilm, releasing the attached cells. Therefore, it was supposed that that the combination therapy of Dep42 and antimicrobial agents may be considered to eliminate dispersed bacterial cells [132].

The examples discussed above concern the activities of phage-derived depolymerases on biofilms under in vitro conditions, in static environments. Such conditions are devoid of human plasma proteins, and they lack in vivo stressors and the response of the immune system. Moreover, in most cases, the experiments did not refer to planktonic infections which usually overlay clinical biofilm infections. Whenever possible, these factors should be included in research as they may have an important role in biofilm eradication and/or prevention.

## 4. Combination Therapy of Phage-Derived Depolymerases and Different Therapeutic Agents

Sometimes, the use of phage-encoded depolymerases as a sole treatment to destroy bacterial biofilms is insufficient. As described above, the Dpo1 of phage Petty can reduce only ~20% of the biofilm biomass [127]. Therefore, it is recommended to use a combination therapy. This approach combines two or more agents simultaneously or sequentially. The combination therapy is composed of some phage-derived depolymerases and different alternative therapeutic agents, including antibiotics, phages, chemical or natural compounds, and detergents (Table 1).

### 4.1. Various Antibiotics

The combination therapy is mainly based on the use of depolymerases together with antibiotics. The synergistic action of free or phage-encoded depolymerases with antibiotics gave optimistic results. The phage-encoded depolymerases not only induced susceptibility of bacteria to antibacterial agents, but also were able to penetrate the biofilm and damage its structure, which is usually not achieved when antibiotics are used alone. The undoubted advantage of this therapy is that phage depolymerases allow degrading EPS/CPS/LPS layers, as well as loosen up and peel off the biofilm structure, thereby allowing antibiotics to easily reach the bacteria and expand more effectively [98,100,113]. As an example, the depolymerase produced by lytic bacteriophage KPO1K2 was used with ciprofloxacin. These studies showed that the combined treatment of depolymerase of phage KPO1K2 and the antibiotic worked more effectively against old biofilms than either of agents used alone [128,129]. In turn, Bansal et al. [134] tested the combinations of phage KPO1K2 or bacterial depolymerase with gentamicin against biofilms formed by *K. pneumonia* strain B5055. In the case of the combined therapy of phage-derived depolymerase and gentamicin, a reduction in bacterial cell number was significant relative to the control variant. Interestingly, this effect was not so spectacular when only gentamicin was used. Moreover, the combination of depolymerase Dep42 with polymyxin was also an effective approach in the fight against bacterial biofilm [132].

### 4.2. Bacteriophages

As both CPSs and EPSs can reduce the efficacy of phage adsorption rate to the surfaces of bacterial cells, the application of whole phage particles, in addition to depolymerases, can increase the phage penetration and expansion within biofilm, thus making it easier for phages to reach the receptors of cells belonging to deeper layers [105,141,142]. Interestingly, phages were also shown to diffuse through alginate exopolysaccharides and cause a reduction in cell number in quite old (20 days) biofilms of *P. aeruginosa* [143]. Another example of such combined therapy is based on the in vitro application of four different agents: (1) lytic phage KP34 with its virion-associated depolymerase KP34p57, (2) the recombinant depolymerase KP34p57, (3) depolymerase-nonbearing lytic phage KP15, and (4) ciprofloxacin against a multidrug-resistant *K. pneumoniae* 77 biofilm [135]. Interestingly, the recombinant depolymerase KP34p57 did not significantly eradicate the biofilm of *K. pneumoniae* 77. However, its effectiveness increased significantly in the presence of the KP15 phage, which indicates that phage-derived proteins with enzymatic activity may be a promising support to depolymerase-nonproducing bacterial viruses. It was also demonstrated that depolymerase KP34p57 did not improve ciprofloxacin activity, giving similar results to antibiotic action itself [135].

### 4.3. Chemical Compounds

Tait et al. [136] tested the effects of treatments of disinfectants and phage enzyme to control *Enterobacter agglomerans* biofilm formation. Polysaccharide depolymerase from bacteriophage φEnt was used with a disinfectant (a nonionic disinfectant, an amphoteric-based disinfectant, or a quaternary ammonium compound). The obtained results showed that the combination of phage φEnt-derived enzyme and a disinfectant was more effective than either of these used alone. Interestingly, the highest efficacy was observed in the combination of phage enzyme with a nonionic disinfectant [136,144].

Another interesting research demonstrated that phage-derived depolymerase could destroy about 80% of bacterial cells from *Klebsiella* biofilm [137]. However, Chai and co-workers also noticed that approximately 92% of the bacterial biofilm was eliminated after pretreatment with this virus enzyme followed by chloride dioxide (ClO_2_) incubation for 30 min. Interestingly, ClO_2_ treatment was less efficient than combination therapy and led to the elimination of only 75% of the *Klebsiella* biofilm [137].

Chhibber et al. [139] tested the activity of a phage enzyme in combination with cobalt sulfate (CoSO_4_) against biofilm formation by *K. pneumoniae* strain B5055. According to observations by Hancock et al. [145], addition of Zn (II) or Co (II) to the substrate (bacterial cells in growth medium) could lead to inhibition of the growth of *E. coli* biofilm. Therefore, in these studies, biofilms were grown in minimal media supplemented with 10 μM FeCl_3_ and CoSO_4_. The results showed that the combination therapy of depolymerase of bacteriophage KPO1K2 and CoSO_4_ completely destroyed the young biofilm (up to 2 days old). This was probably possible due to degradation of the EPS matrix, encompassing the biofilm structure, by the depolymerase of the tested virus that facilitated the diffusion of cobalt ions [139]. Importantly, such satisfactory results were not obtained with the application of depolymerase-nonproducing phage alone, as well as in combination with CoSO_4_ [138].

### 4.4. Natural Compounds

Naturally occurring compounds play essential roles in combination therapy. Chhibber et al. [138] studied the efficacy of phages KPO1K2 (*K. pneumoniae* B5055-specific depolymerase-producing phage), NDP (*K. pneumoniae* B5055-specific depolymerase-nonproducing phage), and Pa29 (*P. aeruginosa* PAO-specific depolymerase-nonproducing phage), in combination with xylitol, in the treatment of *P. aeruginosa* and *K. pneumonia* biofilms. Interestingly, the most efficient reduction of the mixed-species biofilm was observed when the combined therapies of phage KPO1K2, Pa29, and xylitol or phage KPO1K2 and xylitol was used. This can be explained by the depolymerase-producing ability of KPO1K2. It is suggested that the capsular depolymerase of KPO1K2 virus may hydrolyze the polysaccharide layer formed by *K. pneumoniae* on the top of the biofilm structure. Therefore, phage KPO1K2 can interact with the bacterial receptor located on *K. pneumoniae,* thereby causing its lysis. This probably facilitated the penetration of Pa29 and xylitol, leading to disruption of the basal *Pseudomonas* layer [139]. It is worth mentioning that Oliveira et al. [140] demonstrated the synergistic effect of honey and phage EC3a (possessing depolymerase activity) against *E. coli* biofilms. Moreover, they noticed that the effectiveness of this combined therapy is strictly dependent on the type and concentration of honey. Interestingly, the combination of phage EC3a and honey (PF2_25%_) revealed more efficient antibiofilm activity than honey or phage alone. Importantly, the combined strategy of phage and PF2_25%_ prevented the appearance of phage-insensitive mutants [140].

## 5. Conclusions

Currently, more than ever, it is necessary to look for alternative approaches to combat bacterial biofilm. Phage-derived depolymerases appear to be effective in destroying, at least to some extent, biofilms. However, in many cases, the use of these enzymes alone is not enough to eliminate pathogenic bacteria from the treated habitat. Nevertheless, combined treatment with phage depolymerase and antibiotics, chemicals, or natural compounds can probably inhibit/disperse biofilms and be effective in reducing the emergence of resistant mutants, providing a putative approach to combat bacterial infections. It is definitely important to understand mechanisms of the synergy between depolymerases and other agents in destroying bacterial biofilms in order to develop efficient methods allowing to control these complex biological structures. 

## Figures and Tables

**Table 1:** Examples of the combined therapy against bacterial biofilm formation.

Enzyme Name	Agent Used	Biofilm Type	Results (with Regard to the Action of the Agent Alone)	Reference
Depolymerase produced by lytic bacteriophage KPO1K2	Ciprofloxacin	*Klebsiella pneumoniae* strain B5055	Biofilm eradication more pronounced	[124,133]
Depolymerase produced by lytic bacteriophage KPO1K2	Gentamycin	*Klebsiella pneumoniae* strain B5055	Reduction in bacterial counts of young biofilm (up to 4 days)	[134]
Depolymerase Dep42 produced by lytic bacteriophage SH-KP152226	Polymyxin	*Klebsiella pneumoniae* strain 2226	Reduction in bacterial counts	[132]
Depolymerase KP34p57 produced by lytic bacteriophage KP34	Ciprofloxacin	*Klebsiella pneumoniae* strain 77	Reduction in colony counts	[135]
Depolymerase KP34p57 produced by lytic bacteriophage KP34	Depolymerase-nonbearing phage KP15	*Klebsiella pneumoniae* strain 77	Reduction in colony counts	[135]
Depolymerase KP34p57 produced by lytic bacteriophage KP34	Ciprofloxacin together with depolymerase-nonbearing phage KP15	*Klebsiella pneumoniae* strain 77	Reduction in colony counts	[135]
Depolymerase produced by lytic bacteriophage φEnt	Disinfectant	*Enterobacter agglomerans* strain Ent	Biofilm reduction more effective	[136]
Depolymerase obtained from the phage that infects *Klebsiella* strains	Chlorine dioxide	*Klebsiella* sp.	Reduction in biofilm-residing cells	[137]
Depolymerase produced by lytic bacteriophage KPO1K2	Cobalt sulfate	*Klebsiella pneumoniae* strain B5055	Reduction in the bacterial number	[138]
Depolymerase produced by lytic bacteriophage KPO1K2	Xylitol	*Pseudomonas aeruginosa* PAO, *Klebsiella pneumoniae* strain B5055	Biofilm reduction more effective	[139]
Phage EC3a bearing the depolymerase activity	Honey	*Escherichia coli* CECT 434	More efficient antibiofilm activity	[140]

## Data Availability

Not applicable.
